# Genetic diversity, population structure, and selective signature of sheep in the northeastern Tarim Basin

**DOI:** 10.3389/fgene.2023.1281601

**Published:** 2023-11-09

**Authors:** Jieru Wang, Jiajia Suo, Ruizhi Yang, Cheng-long Zhang, Xiaopeng Li, Zhipeng Han, Wen Zhou, Shudong Liu, Qinghua Gao

**Affiliations:** ^1^ College of Life Science and Technology, Tarim University, Alar, Xinjiang, China; ^2^ Key Laboratory of Utilization of Livestock and Forage Resources in Circum-Tarim Region, Ministry of Agriculture and Rural Affairs, Tarim University, Alar, Xinjiang, China; ^3^ College of Animal Science and Technology, Tarim University, Alar, Xinjiang, China; ^4^ Key Laboratory of Tarim Animal Husbandry Science and Technology, Xinjiang Production and Construction Corps, Alar, Xinjiang, China

**Keywords:** sheep, genetic diversity, population structure, selective signature, environmental adaptation

## Abstract

Local sheep in the northeastern Tarim Basin can adapt to dry and low-rainfall regional environments. In this study, three local sheep breeds in the northeastern Tarim Basin, LOP (LOP) sheep, Bayinbuluke (BYK) sheep, and Kunlun (KUN, also known as the Qiemo sheep) sheep, and three introduced sheep breeds, Suffolk (SUF) sheep, Dorset (APD) sheep, and Texel (TEX) sheep, were analyzed for genetic diversity, population structure, and selective signature using the Illumina OvineSNP50K BeadChip. We found that LOP, BYK, and KUN had lower observed heterozygosity and expected heterozygosity than TEX, SUF, and ADP, which were differentiated based on geographic distribution. We performed fixation index (FST) analysis on three local sheep breeds in the northeastern Tarim Basin (LOP, BYK, and KUN) and introduced sheep breeds (TEX, SUF, and ADP) to measure genetic differentiation. Nucleotide diversity (PI) analysis was performed on single-nucleotide polymorphism (SNP) data of LOP, BYK, and KUN. A total of 493 candidate genes were obtained by taking the intersection at a threshold of 5%. Among them, *SMAD2*, *ESR2*, and *HAS2* were related to reproductive traits. *PCDH15*, *TLE4*, and *TFAP2B* were related to growth traits. *SOD1*, *TSHR*, and *DNAJB5* were related to desert environmental adaptation. Analyzing the genetic patterns of local sheep in the northeastern Tarim Basin can protect the germplasm resources of local sheep and promote the development and utilization of sheep genetic resources.

## 1 Introduction

Sheep were domesticated in the Fertile Crescent approximately 11,000 years ago to provide humans with meat, milk, skin, and wool (Zeder, 2008). Adaptation to different agricultural environments and various production goals produced genetic variation in different directions (Kijas et al., 2012). Genome-wide genetic variation markers allow selective intervention in breeding and evaluation at an early stage with great accuracy so that good production traits in sheep can be better transmitted to their offspring (Hayes et al., 2013). Through genome sequencing, Rupp et al. (2015) discovered that *SOCS2* is mediated by the JAK/STAT signaling pathway, which affects inflammatory responses, growth, and milk production. Zhao et al. (2022) identified that the genes *HBB*, *PRDX2*, *GPX1*, *GSTA1*, *COL14A1*, and *LTBP4* contributed to the adaptation of Tibetan sheep to the hypoxic environment of the plateau. Li et al. (2014) identified genes (*TYRP*, *ASIP*, and *MITF*) associated with sheep coat color using a combination of whole-genome association analysis and selective scanning. The study was conducted on 99 Finnsheep with different coat colors (white, black, gray, and brown). Xu et al. (2023) modeled a regulatory network regulating adipose homeostasis in the tail of sheep using a genome-wide selective signature and obtained adipocyte-associated genes, namely, *BMP2*, *PDGFD*, and *VEGFA*. Zhao et al. (2020) screened 73 candidate genes associated with tail fat formation, reproduction, and growth traits using three local Chinese sheep breeds based on the selective signature using the Ovine Infinium HD SNP BeadChip. The Illumina OvineSNP50K BeadChip has been widely used to study genetic diversity and the development of economically important traits in sheep. Ghoreishifar et al. (2019) studied that the genetic diversity of Iranian Zandi sheep is relatively low. Baazaoui et al. (2021) identified candidate genes, such as *CDS2*, *PROKR1*, and *BMP2*, associated with tail fat in sheep from semi-arid regions through the reduction of heterozygosity (ROH) and fixation index (FST) analysis.

The northeastern part of the Tarim Basin, located around the Taklamakan Desert, is characterized by significant temperature fluctuations, long sunlight exposure, low rainfall, and complex terrain (Sweet-Jones et al., 2021). Under these unique geographical and cultural conditions, excellent local breeds (LOP, BYK, and KUN) have emerged. These breeds exhibit traits such as resistance to stress and disease, growth, and reproduction (such as estrus period, ovulation rate, mating conception rate, birth weight of lambs, vitality, and survival rate) even in extreme desert environments, making them highly valuable for research purposes. In this study, genomic selection markers were used to detect genomic differences between three local sheep breeds (LOP, BYK, and KUN) in the northeastern part of the Tarim Basin and three introduced sheep breeds (SUF, APD, and TEX). The aim of this study was to explore the genetic diversity and population structure of local sheep in the Tarim Basin and identify molecular markers that are well suited for adaptation to desert environments. This research reveals the genetic imprints left in the sheep genome, providing a theoretical basis for the conservation and development of local sheep in the Tarim Basin.

## 2 Materials and methods

### 2.1 Animal care

This work was conducted following the specifications of the Ethics Committee of the Tarim University of Science and Technology (SYXK 2020-009).

### 2.2 Animal collection

A total of 465 samples from six sheep breeds were selected for this study. Among them, 62 samples were collected from local sheep in the northeastern Tarim Basin, including 27 KUN samples (from the conservation farm in Zhema County, Bayinbuluke Region, Xinjiang), 20 BYK samples (from the conservation farm in Bayinbuluke Region, Xinjiang), and 15 LOP samples (from the conservation farm in Bayinbuluke, Bayinbuluke Region, Xinjiang), and 403 samples were collected from the International Sheep Genomics Consortium (ISGC) (http://www.Sheephapmap.org), including 152 SUF samples from Australia and Ireland, 148 TEX samples from New Zealand, Germany, and Scotland, and 103 APD samples from Australia.

### 2.3 Genotyping and data quality control

Blood samples were collected using blood collection tubes containing *EDTAK2*, and DNA samples were extracted using a DNA kit (TIANGEN, Beijing, China). The DNA samples were genotyped using the Illumina OvineSNP50 BeadChip at Beijing Compassion Agricultural Science and Technology Co. Quality control of single-nucleotide polymorphism (SNP) genotype data was performed using PLINK v.1.9 to remove unqualified SNPs from the samples (individual detection rate > 0.95, SNP detection rate > 0.95, Hardy–Weinberg equilibrium (HWE) *p-*value ≥ 10^−6^, minor allele frequency (MAF) < 5%, and call rate < 90%).

### 2.4 Genetic diversity and population structure

PLINK v.1.90 (Purcell et al., 2007) was used to calculate the observed heterozygosity (HO), expected heterozygosity (He), and inbreeding coefficient (F). After quality control, principal component analysis (PCA) was conducted on the SNP data. The genetic distances of the six sheep breeds were calculated to observe the clustering of the samples and explore the effects of genetic differences and geographic differences on the populations. VCF2Dis (https://github.com/BGI-shenzhen/VCF2Dis) was used to calculate the P-distance matrix. Based on this matrix, the neighbor-joining tree (NJ tree) phylogenetic relationships were constructed using ATGC: FastME. Such phylogenetic relationships were visualized and analyzed using the iTOL tool (Letunic and Bork, 2016). Ancestral component analysis was performed using ADMIXTURE (Patters et al., 2012), where population stratification can be calculated based on genome-wide genetic variation and the proportion of the genome accounted for by the variation in each individual from each of the K ancestors.

### 2.5 Selective signature

In this study, FST analysis was performed on the SNP data of the three local sheep in the northeastern Tarim Basin (LOP, BYK, and KUN) and introduced sheep breeds (TEX, SUF, and ADP) (in sliding 50-kb windows with 20-kb steps). The FST analysis was performed using VCFtools 0.1.13 (Danecek et al., 2011; Shen et al., 2020). In each comparison, the top 5% genomic regions with the highest scores overlapped were considered to be potential selective signatures.

$$FST=\frac{MSP-MSG}{MSP+\left(n_{c}-1\right)MSG}.$$

Here, *MSP* represents the observed mean square errors for the three local sheep in the northeastern Tarim Basin (LOP, BYK, and KUN) and introduced sheep breeds (TEX, SUF, and ADP), which is calculated as follows:

$$MSP=\frac{1}{S-1}\sum_{i}^{s}n_{i}{\left(P_{Ai}-{\bar{P}}_{Ai}\right)}^{2}.$$

*MSG* represents the observed mean square errors for loci within sheep populations, which is calculated as

$$MSG=\frac{1}{\sum_{i=1}^{S}n_{i}-1}\sum_{i}^{S}n_{i}P_{Ai}\left(1-P_{Ai}\right).$$

In the aforementioned equations, *i* is the subpopulations (where *i* = 1,···, s), *n*_*i*_ is the sample size in subpopulation i, *P*_*Ai*_ is the frequency of the SNP allele *A* in the *i*th subpopulation, and *n*_*c*_ is the mean sample size across samples (Nosrati et al., 2019).

Nucleotide diversity (PI) analysis is an essential measure of population diversity. *PI* was calculated separately for the LOP, BYK, and KUN sheep populations. Finally, the 5% high-ranking values were taken in ascending order to intersect with the FST results. The loci were annotated concerning the sheep genome Ovis Oar_v4.0 (http://www.ncbi.nlm.nih.gov/gene). Gene function annotations were prepared using the NCBI and OMIM databases (http://www.ncbi.nlm.nih.gov/omim).

$$PI=\sum_{j=i}^{S}h_{j},$$

where *S* denotes the number of segregating loci and *h*_*j*_ denotes the heterozygosity of the *j* segregating locus.

### 2.6 Enrichment analysis for candidate genes

First, PI and FST results were selected for crossover analysis and subsequently annotated with the sheep genome Ovis Oar_v4.0 (Conway et al., 2017). Finally, Gene Ontology (GO) enrichment analysis and Kyoto Encyclopedia of Genes and Genomes (KEGG) pathway analysis were performed on the crossover genes using g:Profiler (https://biit.cs.ut.ee/). Among them, GO enrichment was used to predict and elucidate gene products in terms of molecular functions, biological processes, and cellular components (Ashburner et al., 2000). KEGG pathway enrichment was used to identify the major biochemical and signaling pathways in which genes are involved (Kanehisa et al., 2016).

## 3 Results

### 3.1 SNP statistics

A total of 493 individuals and 48,889 SNPs were selected for downstream analysis. Among them, there were 48,198 informative SNPs in the BYK breed, 47,530 informative SNPs in the KUN breed, 48,001 informative SNPs in the LOP breed, 49,873 informative SNPs in the SUF breed, 49,766 informative SNPs in the TEX breed, and 48,795 informative SNPs in the APD breed.

### 3.2 Genetic diversity

Genetic diversity analysis (Table 1) revealed that the three local sheep breeds in the northeastern Tarim Basin, KUN, BYK, and LOP, showed inbreeding coefficients (F) ranging from −0.0906 (KUN) to 0.0072 (BYK), with an average value of −0.0365. *Ho* ranged from 0.5576 (KUN) to 0.5864 (BYK), with a mean value of 0.5695. *He* ranged from 0.5739 (LOP) to 0.5881 (KUN), with a mean value of 0.5819. *F* of sheep breeds in other countries ranged from −0.0384 (APD) to 0.0180 (SUF), with a mean value of −0.0128. *Ho* ranged from 0.6012 (APD) to 0.6160 (TEX), with a mean value of 0.6108. *He* ranged from 0.6088 (SUF) to 0.6223 (TEX), with a mean value of 0.6151.

**TABLE 1:** Location information and genetic diversity of six sheep breeds.

Breed	Collection region	Number (only)	Observed heterozygosity (*Ho*)	Expected heterozygosity (*He*)	Inbreeding coefficient (F)
BYK	Bayinbuluke (China)	20	0.5864	0.5838	0.0072
LOP	Bayinbuluke (China)	15	0.5645	0.5739	−0.0262
KUN (Qiemo)	Bayinbuluke (China)	27	0.5576	0.5881	−0.0906
SUF	Australia	98	0.6153	0.6088	0.0180
	Ireland	54			
APD	Australia	103	0.6012	0.6141	−0.0384
TEX	Germany	46	0.616	0.6223	−0.0180
	Scotland	78			
	New Zealand	24			

### 3.3 Population structure

**FIGURE 1:**
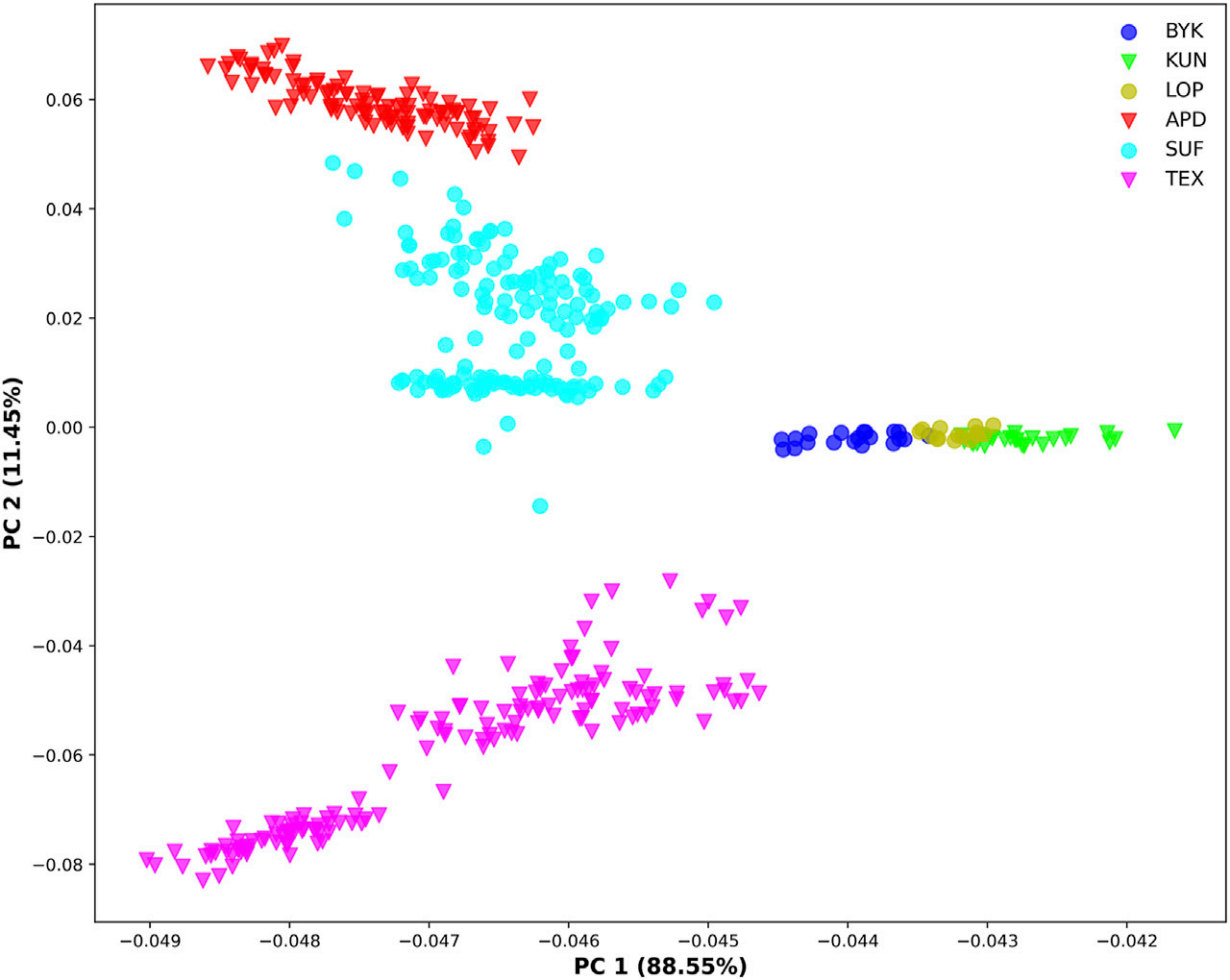
Principal component analysis of six sheep breeds (X-axis represents PC1, and Y-axis represents PC2).

**FIGURE 2:**
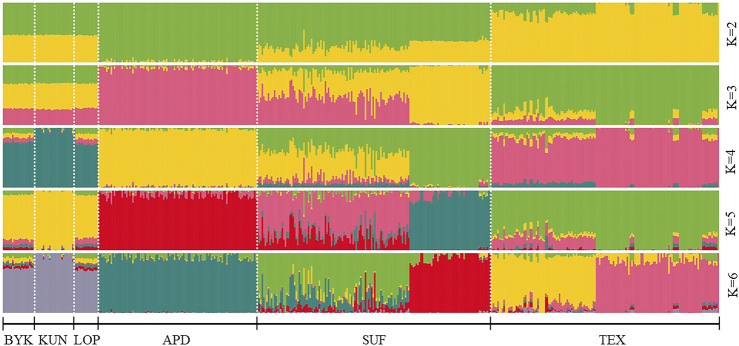
ADMIXTURE analysis of six sheep breeds. The results of the inferred numbers of clusters K = 2–6 are shown. Distinct colors represent different ancestral components.

**FIGURE 3:**
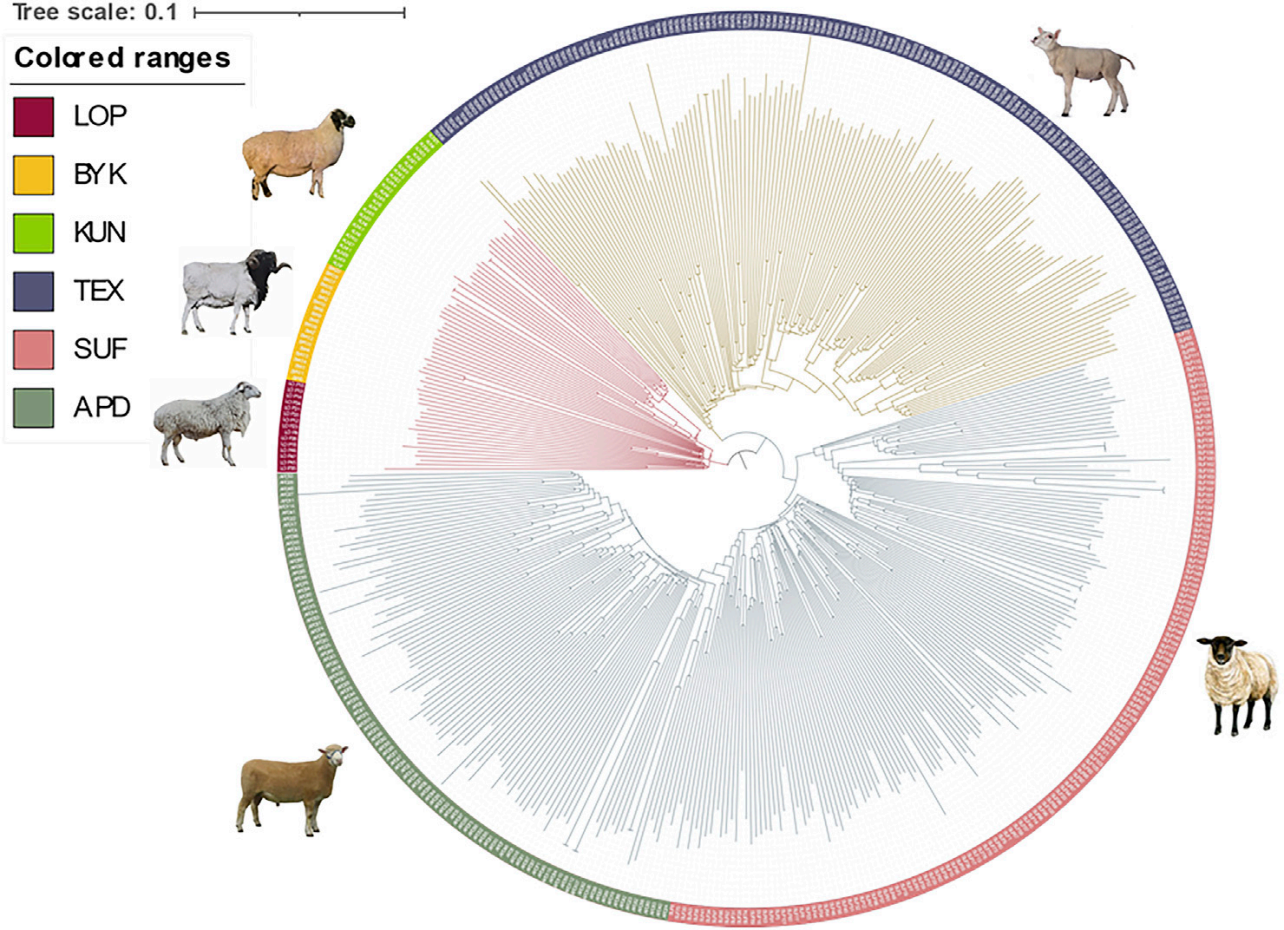
Results of the NJ tree of six sheep breeds. The indigenous sheep breeds in Xinjiang could be distinguished from those in other countries, and the relationships between BYK and LOP were the closest.

The SNP data of 465 sheep were subjected to PCA (Figure 1). The results showed that six sheep breeds were categorized into four subgroups, in which Xinjiang local sheep BYK, KUN, and LOP were clustered together. BTK, LOP and KUN were partially mixed, and KUN extend outwards. Considering the evolutionary process among varieties, we further analyzed the population structure of different populations using ADMIXTURE software (Figure 2). When K = 2–6, LOP, BYK, and KUN sheep all had similar ancestral components, unlike TEX, SUF, and ADP. When K = 6, KUN had a more independent ancestral component, and the BYK and LOP ancestral components were more similar. When K = 6, the Cross Validation (CV) value is the lowest, which can best explain the mixed results. We constructed an NJ tree based on the six sheep breeds (Figure 3). The NJ tree could distinguish between local sheep in the northeastern Tarim Basin and TEX, SUF, and ADP based on the geographic distance, and the results were consistent with the PCA results.

### 3.4 Selective signature

**FIGURE 4:**
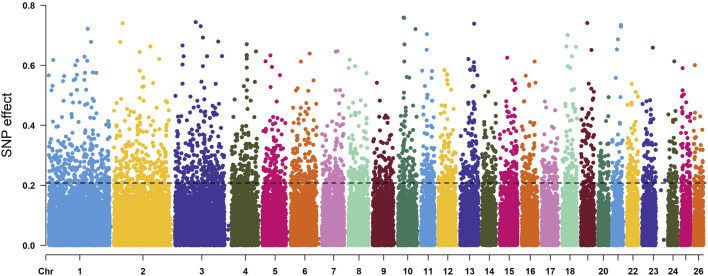
FST detection results for six sheep breeds. The black line represents the 5% threshold line.

**FIGURE 5:**
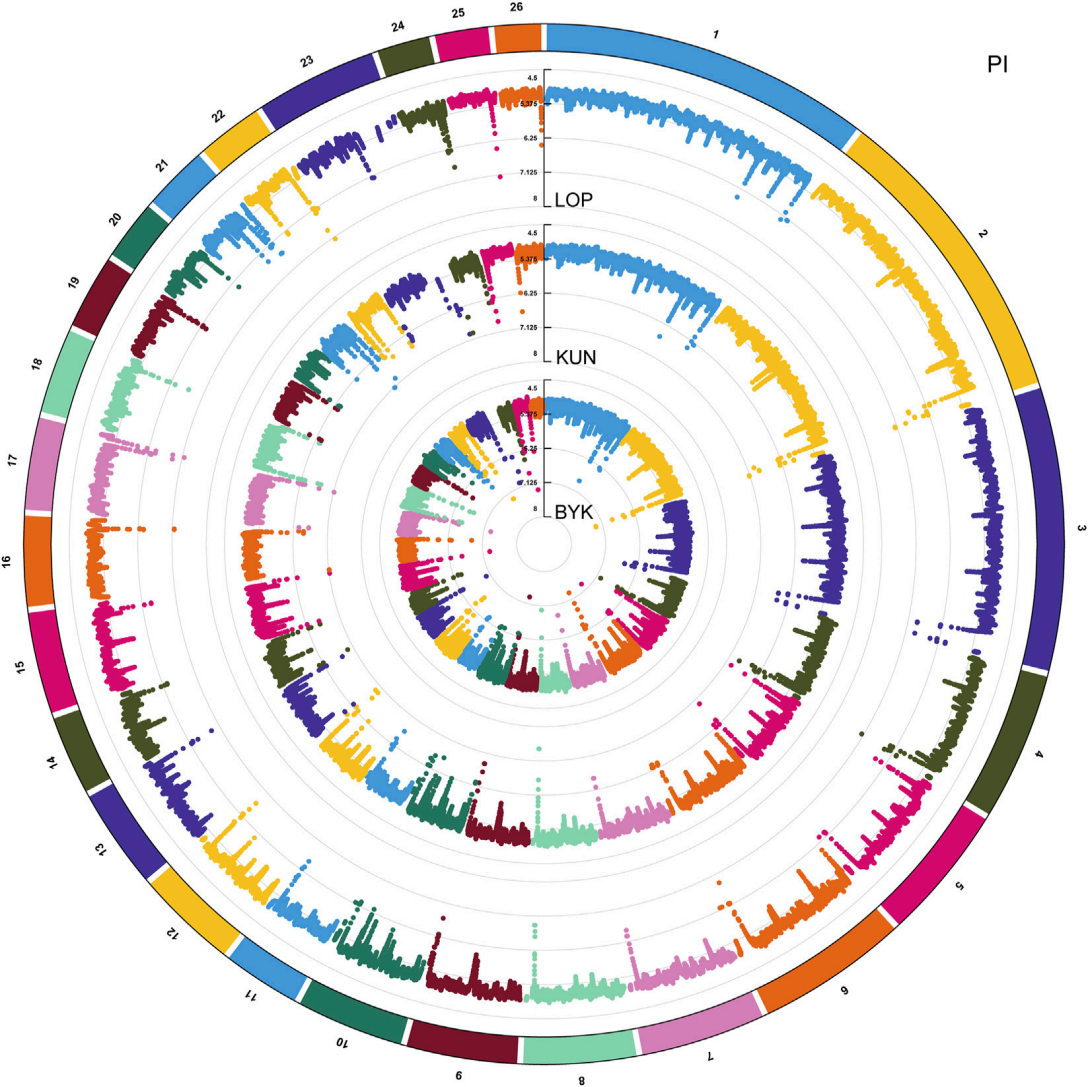
PI analysis results of LOP, BYK, and KUN.

**FIGURE 6:**
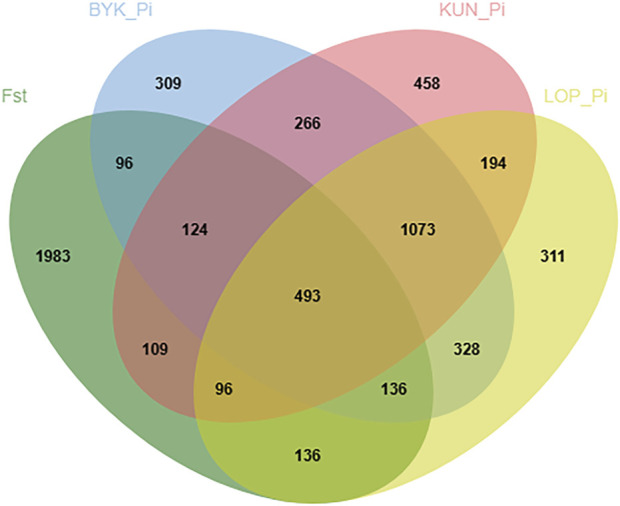
Venn diagrams for KUN, BYK and LOP shared and unique genes.

According to the descending order of FST values, the 5% high-ranking values were regarded as the selection region, and a total of 3,173 genes were obtained after FST screening (Figure 4; Supplementary Table S1). The PI values were in ascending order, and the 5% high-ranking values were regarded as the selection region. The LOP, BYK, and KUN PI values screened 2,825, 2,767, and 2,813 candidate genes, respectively (Figure 5; Supplementary Tables S2–S4). After the deletion of duplicate genes, the genes screened by PI and FST were taken for intersection, and a total of 493 genes were obtained (Figure 6). These genes include *SMAD2*, *ESR2*, and *HAS2*, which are related to reproductive traits. *PCDH15*, *TLE4*, and *TFAP2B* are related to growth traits. *SOD1*, *TSHR*, and *DNAJB5* are related to desert environmental adaptation.

### 3.5 Enrichment analysis for candidate genes

**FIGURE 7:**
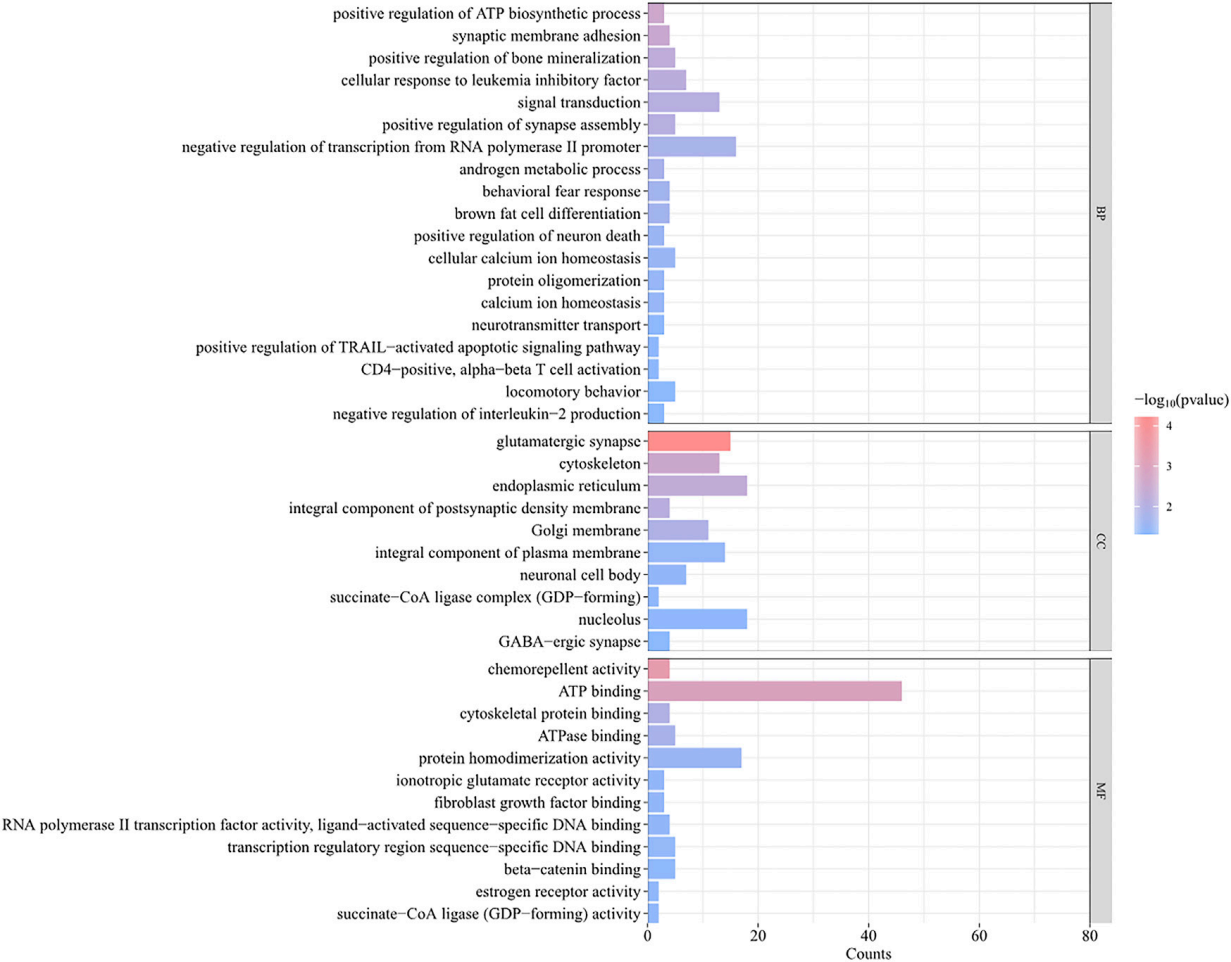
GO analysis of 493 genes obtained by intersection of FST and PI results.

A total of 493 candidate genes were analyzed for GO enrichment and KEGG pathway analyses. GO analysis revealed that 20 genes were enriched in biological processes, 12 genes were enriched in molecular functions, and 10 genes were enriched in cellular components (Figure 7; Supplementary Table S5), including the positive regulation of ATP biosynthetic process, GABAergic synapse, negative regulation of interleukin-2 production, CD4-positive, alpha–beta T-cell activation, negative regulation of transcription from the RNA polymerase II promoter, and chemorepellent activity. The KEGG pathway analysis revealed that eight pathways (Table 2), including metabolic pathways, axon guidance, and cell adhesion molecules, were enriched.

**TABLE 2:** Details of the enrichment pathways of 493 genes obtained by the intersection of FST and PI results.

Category	Term	Genes	FDR
1	Mucin-type O-glycan biosynthesis	*GCNT3*, *GCNT4*, *GALNTL6*, *ST3GAL1*, and *GALNT8*	0.77413
2	Citrate cycle (TCA cycle)	*PDHA2*, *SUCLG2*, *ACO1*, and *SUCLG1*	1
3	Propanoate metabolism	*BCKDHB*, *SUCLG2*, *SUCLG1*, and *HIBCH*	1
4	Arrhythmogenic right ventricular cardiomyopathy	*GJA1*, *SGCD*, *CACNA2D3*, *CACNA1D*, *CTNNA2*, and *ACTB*	1
5	Cell adhesion molecules	*PTPRD*, *NTNG2*, *CDH4*, *CADM1*, *SLITRK1*, *CNTN1*, *CD28*, *SLITRK5*, *CLDN16*, *LRRC4C*, *ICOS*, and *CLDN1*	0.16422
6	Axon guidance	*NTNG2*, *SEMA6A*, *SEMA3D*, *SEMA3A*, *WNT5A*, *CXCR4*, *UNC5C*, *EFNA5*, *SHH*, *RASA1*, *BMPR1B*, *LRRC4C*, and *EPHA3*	0.16422
7	Purine metabolism	*HDDC2*, *PDE10A*, *PDE1A*, *PCDH15*, *TLE4*, *TFAP2B*, *ENPP3*, *PDE7B*, *ADCY8*, *AK8*, and *PGM1*	0.87941
8	Metabolic pathways	*HDDC2*, *PIGO*, *PLOD1*, *HNMT*, *PIK3C2B*, *B3GALT2*, *KYNU*, *PCDH15*, *TLE4*, *TFAP2B*, *MAN1A1*, *ENPP3*, *PGM1*, *HIBCH*, *GALNT8*, *NUDT12*, *CERS6*, *BCKDHB*, *INPP4B*, *UGDH*, *MTHFD1*, *CHDH*, *DPYD*, *SUCLG2*, *SUCLG1*, *PDE1A*, *TYRP1*, *HSD17B4*, *ADCY8*, *LIAS*, *AK8*, *UQCRH*, *INPP5A*, *PRDM16*, *CHST10*, *GCNT3*, *GCNT4*, *MGAT2*, *ST3GAL1*, *KL*, *PDHA2*, *TPK1*, *ASS1*, *SGPP1*, *GALC*, *PDE10A*, *ETNK1*, *ACO1*, *GALNTL6*, *PDE7B*, *LPIN1*, *GCLM*, and *PFKP*	1

## 4 Discussion

### 4.1 Genetic diversity and population structure

We selected the genetic patterns of LOP, BYK, and KUN for analysis to conserve and develop the genetic resources of indigenous sheep germplasms in the northeastern margin of the Tarim Basin. The results showed that the inbreeding coefficients and heterozygosity of the three indigenous sheep in the northeastern margin of the Tarim Basin were lower than those of TEX, SUF, and ADP. The reason for this was that the indigenous sheep lived in the hinterland of the desert environment, where transportation was inconvenient and there was little communication with the outside world. Introducing other breeds was more expensive, leading to less crossbreeding between other sheep breeds and the indigenous sheep of northeastern Xinjiang, resulting in a low degree of heterozygosity. Therefore, we suggest that, in the subsequent breeding of breeds, the local area needs to develop a scientific breeding plan to introduce other good genes to improve genetic diversity. Population structure analysis showed that Xinjiang native sheep were genetically distant from other sheep. The three native sheep in the northeastern margin of the Tarim Basin were close to each other and separated from TEX, SUF, and ADP. The main reason was that LOP, BYK, and KUN lived in desert environments and had long been geographically distant from the introduced sheep breeds. There was a genetic isolation phenomenon, and there were also breed protection effects. PCA and NJ trees could differentiate sheep breeds of different regions according to geographic distances. The results of ADMIXTURE showed that when K = 4–6, BYK and LOP sheep in the northeastern edge of the Tarim Basin are more closely related, mainly because they have similar ancestral components and genetic distances. This result is consistent with the findings of PCA and the evolutionary tree.

### 4.2 Selection sweep methods

The genome-selective sweeping method detects the origin evolution and gene exchange of species (Li et al., 2020) and explores the genetic mechanism of livestock in environmental change. Different sheep breeds have different molecular genetic markers, and molecular markers with specific significance can be obtained using genome-selective sweeping methods. Native sheep in the northeastern margin of the Tarim Basin have environmental adaptations different from those of sheep in other regions living under extreme desert conditions, and their outstanding advantage over other sheep is their resistance to hot temperatures, humidity, disease, parasites, and food shortages. In this study, SNP data of three indigenous sheep breeds in the northeastern Tarim Basin (BYK, LOP, and KUN) and introduced sheep breeds (SUF, APD, and TEX) were analyzed using the genome-selective scanning (GSS) method. PI analysis was performed on BYK, LOP, and KUN. A total of 493 genes were obtained under 5% threshold conditions, and the biology of these genes was revealed. The enrichment results showed genetic evidence and physiological mechanisms for the adaptation of sheep to desert environments in the northeastern margin of the Tarim Basin. The following section discusses only the leading candidate genes and their potential roles in desert adaptation and immune and reproductive traits.

### 4.3 Reproduction-related genes

The reproduction-related trait is one of the most important economic traits in sheep, including follicular development, ovulation, fertilization, embryonic development, and other physiological processes regulated by several factors and genes, and its underlying mechanism is very complex. Sheep from the northeastern margin of the Tarim Basin have undergone natural and artificial selection in the Taklamakan Desert to develop a stable reproductive capacity. Through selective scanning analysis, we identified some candidate genes related to reproduction, including *SMAD2*, *ESR2*, *HAS2*, *DMC1*, *GRM1*, and *TSHR*. *SMAD2* is an important transcription factor downstream of the TGF-β/Smad pathway and is highly expressed in uterine and ovarian tissues. *SMAD2* mediates various physiological processes and is a significant candidate for influencing litter size traits in Tibetan sheep. Genes can be used as molecular genetic markers to assist in selecting improved reproductive characteristics in the initial stages of sheep breeding (Li et al., 2022). Maintaining the survival of dilated oocytes through *SMAD2* improves the fertilization and subsequent development of closed oocytes during oocyte–oocyte complex (*COC*) maturation, thereby enhancing maternal reproductive performance (Wu et al., 2021). Estrogen receptor 2 (*ESR2*) is an essential gene involved in the expression of estrogen receptors, which is expressed in the hypothalamus–pituitary–ovary axis of sheep. The hypothalamus–pituitary–ovarian axis and its expression in single-lamb populations were higher than those in multi-lamb populations. Based on the dose effect of *ESR2*, it was hypothesized that overexpression of the *ESR2* gene in pituitary tissues inhibits LH release, affects follicle development and maturation, and promotes reproductive performance in sheep (Sánchez-Criado et al., 2012; Khristi et al., 2018; Xu et al., 2018). Disrupted meiotic cDNA1 (*DMC1*) controls ovarian meiosis and follicular development in sheep (Mandon-Pépin et al., 2003), and hyaluronan synthase 2 (*HAS2*) is present in dominant follicles in mammals and affects the expansion of the oocyte mound cells to promote oocyte maturation (Zhang et al., 2018). Native sheep utilize these genes, directly and indirectly, to increase reproductive rates and achieve multiple fecundities, which is essential for population continuation in harsh environments. Our study is consistent with that of Luna-Nevárez et al. (2021) who found that the thyroid-stimulating hormone receptor (*TSHR*) gene affects heat tolerance in pregnant Columbia–Rambouillet ewes in a desert environment (Luna-Nevárez et al., 2021). However, there are also unique genetic mechanisms for reproductive traits in native sheep in the Tarim Basin. Liu et al. (2022) identified the *TSHR* gene as a candidate gene for litter size in twin-bearing Pishan sheep in Xinjiang. Ming Li et al. (2023) suggested that the 29-bp nucleotide sequence variation within the *TSHR* gene may be a candidate for improving reproductive traits in sheep through marker-assisted selection (*MAS*). Zhu et al. (2022) also suggested that *GRM1* was the dominant genotype for litter size in Kazakh, Chinese Merino, and Lake sheep and was significantly associated with lambing traits. Native sheep have evolved a more unique and stable reproductive system to ensure pregnancy stability in harsh desert environments.

### 4.4 Fat- and growth-related genes

We also identified some genes related to lipid metabolism, which has been reported to be essential for maintaining energy balance in thermoregulatory responses and is a critical biological process in the immune system under heat stress (Das et al., 2016). Sheep exposure to hyperthermia could regulate body temperature more efficiently and survive heat stress (Romero et al., 2013). Several KEGG pathways of interest related to energy homeostasis and lipid metabolism were significantly enriched in our study, such as energy metabolism and the tricarboxylic acid cycle. These pathways include *CERS6*, *SUCLG2*, *MGAT2*, *PRDM16*, and *PPARGC1A* genes. Ceramide synthase 6 (*CERS6*) alleviates insulin resistance, increases systemic energy expenditure, and maintains glucose homeostasis by improving β-oxidation capacity in liver and brown adipose tissues (Raichur et al., 2019). Succinate-CoA ligase (*SUCLG2*) regulates the conversion of propionate to pyruvate, promotes gluconeogenesis via PGC-1α, and maintains glucose homeostasis in hepatic gluconeogenesis (Wang et al., 2022a). Monoacylglycerol acyltransferase 2 (*MGAT2*) is a crucial enzyme highly expressed in the human small intestine and liver and regulates triglyceride uptake and homeostasis (Cheng et al., 2022). The peroxisome proliferator-activated receptor gamma coactivator 1 alpha (*PPARGC1A*) has been associated with obesity and related metabolic disorders, affecting adipocyte differentiation and lipid metabolism (Zhang et al., 2022). In practice, these adaptive genes induce energy homeostasis in response to relatively high energy demands under heat stress conditions. They regulate hypothermia caused by significant diurnal and nocturnal colds, making it easier for sheep to adapt to harsh desert environments.

Our study also identified some genes related to meat production. Zlobin et al. (2023) identified protocadherin 15 (*PCDH15*), a gene associated with meat productivity, between domesticated sheep (*Ovis aries*) and pan sheep (*Ovis ammon*), which is consistent with a super-dominant model of inheritance. Kizilaslan et al. (2022) studied the genetic structure of growth- and linear-type traits in Akkaraman sheep and proposed 22 genes, among which transducin-like enhancer of split-4 (*TLE4*) and transcription factor AP-2 β (*TFAP2B*) were same as the genes found in this study (Kizilaslan et al., 2022). Abousoliman showed that the activation rate of muscle stem cells is determined by the level of paired box 3 (*Pax3*), and alternative polyadenylation of *Pax3* controls muscle stem cell fate and muscle function (de Morree et al., 2019). Genome-wide analysis for early growth-related traits of the locally adapted Egyptian Barki sheep revealed 10 genes related to early growth traits, among which *SLC16A7* and *TFAP2B* were same as the genes found in our study (Abousoliman et al., 2021).

### 4.5 Genes related to desert adaptation

Recently, it has been shown that functional genes play a role in epigenetic temperature regulation mechanisms that lead to heat adaptation-mediated adaptive behaviors and protective responses in cells (Akerman et al., 2016) and that the foremost adaptation of the desert environment is the heat stress response. Four genes associated with heat stress were identified in our study. Superoxide dismutase 1 (*SOD1*) is a gene that regulates heat tolerance and maintains cellular oxidative homeostasis from superoxide radicals generated by stress in desert environments, such as heat and humidity (Khan et al., 2020). The DnaJ (*Hsp40*) homolog subfamily B member 5 (*DNAJB5*) gene affects heat tolerance in Dolan sheep, which is consistent with our study results. The *DNAJB5* gene chaperone complex controls polarized growth by repressing Hsf1-driven heat stress-associated transcription (Vjestica et al., 2013; Wang et al., 2019). The *TSHR* gene regulates HSPs, which are believed to function as cellular thermometers for environmental adaptation in heat-stressed small ruminants (Luna-Nevárez et al., 2021). The phosphatidylinositol-4-phosphate 3-kinase catalytic subunit type 2 beta (*PIK3C2B*) gene is categorized in a cluster of genes compatible with the PI3K isoform. This subtype induces energetic homeostasis in response to high energy demands under heat stress conditions and is significantly related to avian mechanisms of acclimation to heat stress (Kim et al., 2017). Xie et al. (2023) concluded that the *PIK3C2B* gene is involved in glycerophospholipid biosynthesis, lipid modification, phospholipid biosynthesis, and phosphatidylinositol metabolism. It is more abundantly expressed in the function and pathway of lipids, and the *PIK3C2B* gene is one of the critical genes affecting fat deposition or lipid metabolism of swine (Xie et al., 2023). By combining the aforementioned ideas, we suggest that the *PIK3C2B* gene may maintain energy homeostasis by affecting fat metabolism, maintaining energy balance, and regulating body temperature, which in turn enables sheep to adapt better to the heat stress environment and maintain energy homeostasis.

Sheep are subjected to various diseases and parasites in their natural wild environment. Our study detected that genes such as *CCL26*, *CTLA-4*, *PTPN13*, *PKHD1*, *ICOS*, and *ARPP21* are associated with immunity, and genes such as *WNT5A*, *TIMP3*, and *BIN1* are associated with parasite resistance. C–C ligand 26 (*CCL26*) protects against localized infections, participates in the immune response, and plays a role in resistance to tick infestation and disturbance (Thutwa et al., 2021). Mature eosinophils are activated by *CCL26* and migrate to the infection site, acting as an immunizing agent (E et al., 2019). In *in vitro*, when B7 molecules on the surface of antigen-presenting cells bind to cytotoxic T-lymphocyte-associated antigen 4 (*CTLA-4*) on the surface of T cells, a cost stimulus signal for T-cell activation is generated, which affects the humoral immune response in the body (Linsley et al., 1992). In a study on lambs in Spain, it was found that temperature and humidity affected the infectivity of *Mycoplasma*, with higher temperatures and lower humidity resulting in higher infection rates. This is quite similar to our environment, and the same protein tyrosine phosphatase non-receptor 13 (*PTPN13*) gene has been found in the immune function pathway (Fernández et al., 2016; Mousel et al., 2021). Polycystic kidney and liver disease 1 (*PKHD1*), inducible T-cell co-stimulatory factor (*ICOS*), and cAMP-regulated phosphoprotein 21 (*ARPP21*) enhance mastitis resistance. Innate and acquired immune responses trigger the production of T-assisted type 2 cytokines (*Th2*), which participate in the immune response. Benavides et al. described that they play multiple roles in innate and acquired immune response mechanisms and cytokine signaling, engage in hemostatic regulation and mucosal defense, and are essential for protecting sheep against parasitic invasion (Benavides et al., 2016; Banos et al., 2017; Gutiérrez-Gil et al., 2018; Öner et al., 2021).

## 5 Conclusion

In this study, we analyzed the genetic patterns and genomic differences between native sheep breeds in the northeastern Tarim Basin and introduced sheep breeds by genome-selective scanning. In addition, a genomic selection signaling map of the native sheep population in the Tarim Basin was constructed, revealing the genes and their network regulatory mechanisms associated with stress and disease resistance, growth, and reproductive traits. This study will help improve the production capacity of sheep and provide theoretical support for the conservation and development of sheep germplasm genetic resources in extreme desert environments.

## Data Availability

The data presented in the study are deposited in the Figshare repository, accession number http://doi.org/10.6084/m9.figshare.24461143.
