# Corrigendum: Genetic diversity, population structure, and selective signature of sheep in the northeastern Tarim Basin

**DOI:** 10.3389/fgene.2023.1336294

**Published:** 2023-11-29

**Authors:** Jieru Wang, Jiajia Suo, Ruizhi Yang, Cheng-long Zhang, Xiaopeng Li, Zhipeng Han, Wen Zhou, Shudong Liu, Qinghua Gao

**Affiliations:** ^1^ College of Life Science and Technology, Tarim University, Alar, Xinjiang, China; ^2^ Key Laboratory of Utilization of Livestock and Forage Resources in Circum-Tarim Region, Ministry of Agriculture and Rural Affairs, Tarim University, Alar, Xinjiang, China; ^3^ College of Animal Science and Technology, Tarim University, Alar, Xinjiang, China; ^4^ Key Laboratory of Tarim Animal Husbandry Science and Technology, Xinjiang Production and Construction Corps, Alar, Xinjiang, China

**Keywords:** sheep, genetic diversity, population structure, selective signature, environmental adaptation

In the published article, there was an error in **Affiliations 1, 2, 3**. Instead of “Jieru Wang^1,2,3^, Jiajia Suo^1^, Ruizhi Yang^1^, Cheng-Long Zhang^2,3^, Xiaopeng Li^2,3^, Zhipeng Han^2,3^, Wen Zhou^2,3^, Shudong Liu^2,3,^* and Qinghua Gao^1,2,3,^*; ^1^Key Laboratory of Protection and Utilization of Biological Resources in Tarim Basin Co-Funded by Xinjiang Production and Construction Corps and The Ministry of Science and Technology, College of Life Science and Technology, Tarim University, Alar, Xinjiang, China; ^2^Key Laboratory of Tarim Animal Husbandry Science and Technology of Xinjiang Production and Construction Corps, College of Animal Science and Technology, Tarim University, Alar, China; ^3^Livestock and Forage Resources in Circum-Tarim Region, Ministry of Agriculture and Rural Affairs, Tarim University, Alar, China,” it should be “Jieru Wang^1,2^, Jiajia Suo^1^, Ruizhi Yang^1^, Cheng-long Zhang^3,4^, Xiaopeng Li^3,4^, Zhipeng Han^3,4^, Wen Zhou^3,4^, Shudong Liu^2,3,4,^* and Qinghua Gao^2,3,4,^*; ^1^College of Life Science and Technology, Tarim University, Alar, Xinjian, China; ^2^Key Laboratory of Utilization of Livestock and Forage Resources in Circum-Tarim Region, Ministry of Agriculture and Rural Affairs, Tarim University, Alar, Xinjiang, China; ^3^College of Animal Science and Technology, Tarim University, Alar, Xinjiang, China; ^4^Key Laboratory of Tarim Animal Husbandry Science and Technology, Xinjiang Production and Construction Corps, Alar, Xinjiang, China.”

Furthermore, there was an error in the **Conflict of interest** statement. Instead of “The authors declare that the research was conducted in the absence of any commercial or financial relationships that could be construed as a potential conflict of interest.” it should be “Authors C-lZ, XL, ZH, WZ, SL, and QG were employed by Xinjiang Production and Construction Corps.

The remaining authors declare that the research was conducted in the absence of any commercial or financial relationships that could be construed as a potential conflict of interest.”

The authors apologize for these errors and state that this does not change the scientific conclusions of the article in any way. The original article has been updated.

